# Clinical Assessment and Genetic Testing for Hereditary Polyposis Syndromes in an Italian Cohort of Patients with Colorectal Polyps

**DOI:** 10.3390/cancers16213617

**Published:** 2024-10-26

**Authors:** Candida Fasano, Filomena Cariola, Giovanna Forte, Antonia Lucia Buonadonna, Paola Sanese, Andrea Manghisi, Martina Lepore Signorile, Katia De Marco, Valentina Grossi, Vittoria Disciglio, Cristiano Simone

**Affiliations:** 1 Medical Genetics, National Institute of Gastroenterology, IRCCS “Saverio de Bellis” Research Hospital, 70013 Castellana Grotte, Italy; candida.fasano@irccsdebellis.it (C.F.); filo.cariola@irccsdebellis.it (F.C.); giovanna.forte@irccsdebellis.it (G.F.); lucia.buonadonna@irccsdebellis.it (A.L.B.); paola.sanese@irccsdebellis.it (P.S.); andrea.manghisi@irccsdebellis.it (A.M.); katia.demarco@irccsdebellis.it (K.D.M.); valentina.grossi@irccsdebellis.it (V.G.); 2 Medical Genetics, Department of Precision and Regenerative Medicine and Jonic Area (DiMePRe-J), University of Bari Aldo Moro, 70124 Bari, Italy

**Keywords:** hereditary polyposis syndromes, clinical eligibility criteria, genetic testing, *APC*, *BMPR1A*, *MUTYH*, *SMAD4*, *STK11*, pathogenic variant (PV), variant of uncertain significance (VUS)

## Abstract

**Background**: Hereditary polyposis syndromes are clinically and genetically heterogeneous conditions associated with increased colorectal cancer risk. They are classified based on polyp histology, inheritance mode, causal gene, and colonic and extracolonic manifestations. Their diagnosis is challenging due to overlapping and heterogeneous clinical presentations. **Methods**: A multigene next-generation sequencing panel was used to screen 75 index cases with colorectal polyps and a personal/family history of cancer for key hereditary polyposis-associated genes (*APC*, *BMPR1A*, *MUTYH*, *PTEN*, *SMAD4*, and *STK11*) in order to identify germline genetic variants. **Results**: In the screened index cases, we found 14 pathogenic variants involving *APC*, *MUTYH*, *SMAD4*, and *STK11* and 6 variants of uncertain significance involving *APC*, *BMPR1A*, and *SMAD4*. In this cohort, four patients not fulfilling the recommended eligibility criteria of current National Comprehensive Cancer Network (NCCN) guidelines for genetic testing were molecularly diagnosed with a hereditary polyposis syndrome. **Conclusions**: Our findings indicate that stringent NCCN eligibility criteria for molecular screening may lead to missing some of the patients affected by hereditary polyposis syndromes. This highlights the need for a careful evaluation of patients’ clinical manifestations, polyp number, age of polyp onset, and family history to select appropriate candidates for molecular diagnosis of these conditions.

## Simple Summary

This study reports the results of genetic testing to identify germline variants in the main genes (*APC*, *BMPR1A*, *MUTYH*, *PTEN*, *SMAD4*, *STK11*) associated with hereditary polyposis syndromes in 75 index cases with colorectal polyps and a personal/family history of cancer that had been referred to genetic counseling at the Medical Genetics Unit of the National Institute of Gastroenterology “Saverio de Bellis”, Castellana Grotte, Bari, Italy. In the screened patients, some of which did not meet the recommended eligibility criteria of current National Comprehensive Cancer Network (NCCN) guidelines for genetic testing, we identified 14 pathogenic variants and 6 variants of uncertain significance. Of note, by combining the results of multigene panel tests with the evaluation of patients’ clinical phenotype and family history, we were able to confirm the diagnosis of hereditary polyposis syndrome for pathogenic variant carriers and assign them to specific clinical surveillance and management programs.

## 1. Introduction

Colorectal cancer (CRC) is the third most frequently diagnosed cancer and the second leading cause of cancer death worldwide [1]. While sporadic disease accounts for the vast majority of CRC cases, around 5% of all cases are associated with inherited predisposition due to germline pathogenic variants (PVs) in high-risk cancer genes. Additionally, up to 30% of patients have a family history of CRC, likely related to a combination of inheritance and common environmental risk factors [2]. Inherited CRC predisposition syndromes can be classified based on the presence or absence of polyposis [3]. Hereditary non-polyposis colorectal cancer (HNPCC), also known as Lynch syndrome (LS), is caused by germline PVs in mismatch repair (MMR) genes [4]. In contrast to LS, hereditary polyposis syndromes are widely heterogeneous, both clinically and genetically. They can be classified based on histologic characterization of polyps (adenomatous, hamartomatous, serrated, or mixed polyposis), inheritance mode (dominant or recessive), involvement of specific regions of the gastrointestinal (GI) tract or extraintestinal organs and tissues, and underlying causal gene [5]. The most common and well-characterized condition associated with hereditary polyposis and CRC is familial adenomatous polyposis (FAP). FAP is an autosomal dominant condition caused by *APC* germline alterations and is characterized by the development of numerous colorectal adenomatous polyps and an increased risk of developing CRC [6]. Based on the number of colorectal polyps (CPs), age of CP onset, and extracolonic manifestations, *APC*-associated polyposis includes two main clinical phenotypes, i.e., classical FAP, which is characterized by the early onset of more than 100 colorectal adenomatous polyps, and attenuated FAP (AFAP), which is characterized by the development of fewer colorectal adenomatous polyps (<100) and a later onset of clinical manifestations [6]. Recently a novel FAP clinical variant, termed gastric polyposis and desmoid FAP (GD-FAP), which is associated with profuse gastric polyposis or adenomas, a higher risk of developing desmoid tumors, and less than 50 CPs, has been described [7]. Other hereditary polyposis syndromes include *MUTYH*-associated polyposis (MAP) and a group of hamartomatous polyposis syndromes (HPSs). MAP is inherited in an autosomal recessive manner and is caused by biallelic mutations in the *MUTYH* gene [8]. As with FAP, patients with MAP may develop colorectal adenomatous polyps and have an increased lifetime risk of GI cancers [9]. HPSs include Peutz–Jeghers syndrome (PJS), which is caused by germline alterations in the *STK11* gene, hereditary juvenile polyposis (JPS), which is caused by germline alterations in the *BMPR1A* and *SMAD4* genes, and PTEN hamartoma tumor syndrome (PHTS), which is caused by germline alterations in the *PTEN* gene [10]. The germline PVs involving the well-known genes *(APC*, *BMPR1A*, *MUTYH*, *PTEN*, *SMAD4*, and *STK11*) impact several signaling pathways (Wnt signaling, TGF-β signaling, DNA repair signaling, and the PI3K/AKT pathway) that modulate the carcinogenesis processes of CRC [11].

The diagnosis of these syndromes in their early stage is complicated by the presence of milder phenotypic manifestations, such as a small number of polyps, which are often not well defined histologically. Although relatively expensive in terms of costs and time, genetic testing is a critical tool to identify PVs in the main genes associated with hereditary polyposis syndromes. To ensure effective management of healthcare resources, clinical geneticists should therefore carefully evaluate the eligibility criteria of probands for molecular testing based on the presence or absence of phenotypic manifestations, personal and family history of GI polyposis, and CRC, as indicated by clinical guidelines. According to current guidelines from the National Comprehensive Cancer Network (NCCN), genetic testing for high-risk genes associated with hereditary polyposis syndromes (*APC*, *MUTYH*) is recommended in individuals with 20 or more colorectal adenomatous polyps and/or a known familial PV in these genes and/or a family history of polyposis, while it may be considered in individuals with 10–19 colorectal adenomatous polyps, desmoid tumor, hepatoblastoma, or individuals who meet the criteria for serrated polyposis syndromes (more than five serrated polyps) with at least some adenomatous polyps. Additionally, patients with greater than or equal to two hamartomatous polyps are recommended to undergo genetic testing for the main genes associated with JPS, PJS, or PHTS (*BMPR1A*, *PTEN*, *SMAD4*, *STK11*) [12].

Overall, the presence of inherited pathogenic germline variants in the abovementioned genes promotes carcinogenesis, with precursor CPs developing into CRC [13]. The identification of a germline PV in these high-risk genes in individuals with familial CPs and/or CRC is crucial to enable tailored surveillance and appropriate risk reduction interventions [5,14].

In the present retrospective study, we report on our seven-year experience evaluating families with CPs and a history of cancer. All the patients included in this study were screened for genetic variants in the main genes associated with hereditary polyposis syndromes (*APC*, *BMPR1A*, *MUTYH*, *PTEN*, *SMAD4*, *STK11*), which allowed us to identify germline PVs and variants of uncertain significance (VUSs). Patients carrying PVs or VUSs in these genes were further characterized by analyzing in detail their clinical phenotypes and family history.

## 2. Materials and Methods

### 2.1. Patient Selection

A total of 75 index cases with CPs and a personal/family history of cancer evaluated at the Medical Genetics Unit of the National Institute of Gastroenterology “Saverio de Bellis”, Castellana Grotte, Bari, Italy, between January 2017 and July 2024 were included in this study. Case and family history were collected, including clinicopathological and demographic information, and a pedigree was generated for each family. Among the index cases with CRC, we only included those with an MMR-proficient phenotype. CRC patients meeting the criteria of a screening algorithm for LS, i.e., CRC showing MMR loss/high microsatellite instability (MSI-H) and wild-type genotype for BRAF^V600^, were excluded from the present study as they were described in a previous report [15]. Briefly, the fully automated Idylla^TM^ MSI Test was used to detect microsatellite status from colorectal cancer tissue following the manufacturer’s instructions (Biocartis, Mechel, Belgium). This molecular test uses a PCR reaction and high-resolution melting curve analysis to analyze seven loci (*ACVR2A*, *BTBD7*, *DIDO1*, *MRE11*, *RYR3*, *SEC31A*, and *SULF2*) to determine the microsatellite status in CRC. A sample’s microsatellite status has been ascertained via automated software interpretation and reporting. Additionally, the detection of the wild-type or V600E-mutated BRAF was performed following the manufacturer’s instruction of the Idylla BRAF Mutation Test Kit (Biocartis, Mechel, Belgium). Written informed consent to perform molecular testing on blood and/or pathological tissue specimens was obtained from the patients and their relatives using a form approved by a competent ethics committee, in line with the principles of the Declaration of Helsinki and any other applicable local ethical and legal requirements (protocol code N_170, date of approval 31 October 2016). Moreover, blood samples were collected from the index cases and their consenting family members for prospective genetic testing.

### 2.2. Genetic Testing

Genomic DNA was extracted from peripheral blood with the QIAamp DNA Blood Mini Kit (Qiagen, Hilden, Germany) according to the manufacturer’s instructions. The complete coding region, exon–intron boundaries, and flanking sequences of major hereditary polyposis-associated genes (*APC*, *BMPR1A*, *MUTYH*, *PTEN*, *SMAD4*, and *STK11*) were screened for the presence of genetic variants using a multigene next-generation sequencing (NGS) panel, followed by detection of copy number variation (CNV). The analysis was performed as previously described [15]. Briefly, libraries were generated from 10 ng of whole-blood DNA using the Ion AmpliSeq targeted sequencing technology on an Ion Chef System (Thermo Fisher Scientific, Waltham, MA, USA). Clonal amplification and chip loading of the libraries were performed on an Ion Chef System (Thermo Fisher Scientific, Waltham, MA, USA). The libraries were sequenced on an Ion GeneStudio S5 Prime System (Thermo Fisher Scientific, Waltham, MA, USA) using specific kits according to the manufacturer’s instructions. Reads were aligned to the hg19 human reference genome, and data analysis was carried out using Torrent Suite Software v.5.12.1 (Thermo Fisher Scientific, Waltham, MA, USA). The identified genetic variants putatively responsible for the clinical phenotype of the index cases were validated using Sanger sequencing. Primer sequences are available upon request. CNV analysis was performed on whole blood-extracted DNA by multiplex ligation-dependent probe amplification (MLPA) using the SALSA MLPA P043 APC, P158 JPS, P378 MUTYH, P225 PTEN, and P101 STK11 kits (MRC Holland, Amsterdam, The Netherlands) according to the manufacturer’s instructions. The amplification products were separated on an ABI Prism 3130 Genetic Analyser (Thermo Fisher Scientific, Waltham, MA, USA). The migration of fragments was calculated by comparison to the GeneScan LIZ-500 size standard (Thermo Fisher Scientific, Waltham, MA, USA). Data analysis was performed using Coffalyser software v.220513.1739 (MRC Holland, Amsterdam, The Netherlands).

### 2.3. Variant Classification

The clinical significance of each variant was annotated using the American College of Medical Genetics and Genomics/Association for Molecular Pathology (ACMG/AMP) guidelines [16], as well as the pathogenicity assertions registered in ClinVar.

### 2.4. In Silico Prediction Analysis of Variants of Uncertain Significance

In silico pathogenicity prediction analysis of missense VUSs was performed using Panther db (Protein ANalysis THrough Evolutionary Relationships; https://www.pantherdb.org/, accessed on 28 June 2024), PolyPhen-2 (Polymorphism Phenotyping v2; http://genetics.bwh.harvard.edu/pph2/, accessed on 28 June 2024), and SIFT (Sorting Intolerant From Tolerant, https://sift.bii.a-star.edu.sg, accessed on 28 June 2024). Panther db is a free web resource that predicts potential functional alterations in proteins upon single amino acid substitutions based on protein phylogeny [17]. In addition to assessing the potential effects of genetic variations on specific amino acid positions, this database provides a range of computational methods to analyze the functional properties of genes, conduct enrichment analysis, and perform homology annotation [18]. PolyPhen-2 uses an algorithmic approach developed to predict how protein structure and function are affected by amino acid substitutions. This in silico prediction method is based on a probabilistic classifier that calculates the probability of each specific amino acid substitution impacting different structural, phylogenetic, and sequence features [19]. The SIFT algorithm relies on a position-specific scoring matrix (PSI-BLAST) method to assess the potential deleteriousness of a missense variant by examining sequence similarity [20]. SIFT effectively predicts deleterious mutations in conserved protein regions by considering the precise location and characteristics of the amino acid modification [20]. All in silico prediction evaluations were conducted using default parameter settings.

## 3. Results

### 3.1. Genetic Testing Results

**Figure 1:**
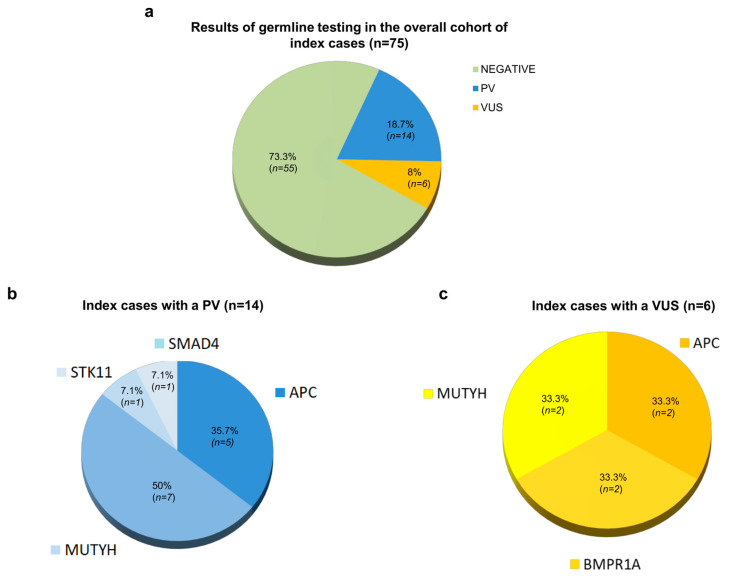
Overview of the genetic testing results of the 75 index cases included in this study. (**a**) Percentage distribution of the index cases with a pathogenic variant (PV), a variant of uncertain significance (VUS), or no identified variant. (**b**) Percentage distribution of the 14 index cases carrying a PV in a hereditary polyposis-associated gene (*APC*, *MUTYH*, *SMAD4*, and *STK11*). (**c**) Percentage distribution of the 6 index cases carrying a VUS in a hereditary polyposis-associated gene (*APC*, *BMPR1A*, and *MUTYH*).

A total of 75 index cases with CPs and a personal/family history of cancer were analyzed to identify genetic alterations in major hereditary polyposis-associated genes (*APC*, *BMPR1A*, *MUTYH*, *PTEN*, *SMAD4*, *STK11*). No CNV event was detected in the analyzed genes in our study cohort. Moreover, this genetic testing revealed that 14 (18.7%) index cases had a germline PV in *APC*, *MUTYH*, *SMAD4*, or *STK11*, thus meeting the diagnostic criteria for hereditary polyposis syndromes, 6 (8%) had a VUS in *APC*, *BMPR1A*, or *MUTYH*, and 55 (73.3%) did not harbor genetic alterations involving the analyzed genes (Figure 1a).

Among the 14 index cases with hereditary polyposis syndromes, 5 different *APC* PVs were found in 5 (35.7%) individuals; 7 different *MUTYH* PVs were found in homozygous/compound heterozygous state in 7 (50%) individuals; 1 *SMAD4* PV was found in 1 (7.1%) individual; and 1 *STK11* PV was identified in 1 (7.1%) individual (Figure 1b, Table S1, Figures S1a,b and S2a,b). Furthermore, among the 6 index cases with a VUS, 2 different *APC* VUSs were found in 2 (33.3%) individuals, 2 different *BMPR1A* VUSs were found in 2 (33.3%) individuals, and 2 different *MUTYH* VUSs were found in a heterozygous state in 2 (33.3%) individuals (Figure 1c, Table S2, Figures S1a,b and S2c).

### 3.2. Clinical and Molecular Findings of Patients with PVs in Hereditary Polyposis Syndrome-Associated Genes

Among the 75 index cases included in this study, 12 (16%) were molecularly diagnosed with an adenomatous polyposis syndrome (5 FAP [6.7%], 7 MAP [9.3%]), 1 (1.3%) with JPS, and 1 (1.3%) with PJS based on genetic testing. Of note, genetic testing for the identification of PVs in major adenomatous polyposis-associated genes (*APC*, *MUTYH*) was also performed in index cases with 10–19 colorectal adenomatous polyps (and their relatives), for which this screening may be considered but is not a recommendation based on current NCCN guidelines (version 1.2024) [12].

In accordance with NCCN guidelines, the identified *APC* and *MUTYH* PVs were segregated in the carrier index cases’ family members willing to undergo genetic testing. Phenotypically, among all FAP/MAP-affected index cases and family members, 14 individuals (9 index cases and 5 family members) had 20 or more colorectal adenomatous polyps, 4 individuals (3 index cases and 1 family member) had between 10 and 19 colorectal adenomatous polyps, and 1 family member had less than 10 colorectal adenomatous polyps.

In family 1, a heterozygous *APC* PV (c.225dupT, p.Asn76Ter) was identified in a female patient who developed between 10 and 19 colorectal adenomatous polyps and CRC at 59 years of age, in the absence of a family history of CPs, CRC, or FAP-associated malignancy. The identified *APC* c.225dupT PV was segregated in the index case’s family members, revealing that her 38- and 34-year-old daughters inherited the variant although they did not exhibit clinical manifestations. This family and the identified variant were analyzed clinically and molecularly in a previous report by our group [21].

In family 2, a splice donor site mutation in *APC* exon 12 (c.1621_1626+7del, p.A517_Q542del) leading to exon 12 skipping was identified in a 53-year-old male patient who developed ≥20 colorectal adenomatous polyps and had a family history of colorectal polyposis and CRC. This family and this *APC* variant were also characterized in detail in a previous study by our group [21,22].

In family 3, the index case was a female patient who developed multiple colorectal adenomatous polyps (≥20) and gastric polyps and had a family history of CPs, CRC, and FAP-associated malignancies. Genetic testing revealed a heterozygous G deletion at position c.2162 of the *APC* gene resulting in a premature stop codon and a putative truncated protein (c.2162delG, p.Gly721Glufs*6). The identified *APC* PV was also detected in the index case’s mother, who developed CRC at 30 years of age.

In family 4, a germline PV in the *APC* gene (c.2805C>A, p.Tyr935Ter) was identified in a 34-year-old female patient with a classical FAP phenotype who developed CRC and multiple CPs. Her family history could not be collected as the patient was adopted.

In family 5, concurrent germline PVs in *APC* (c.1111G>T, p.Gly371*) and *MUTYH* (c.536A>G, p.Tyr179Cys) were identified in individuals who had CPs and CRC [23]. In this family, the index case and three of her family members only harbored the *APC* variant, three other family members only harbored the monoallelic *MUTYH* variant, and the remaining two concurrently harbored both [23]. Among the individuals carrying only the *APC* PV, the index case and her maternal uncle developed ≥ 20 colorectal adenomatous polyps, while the index case’s mother developed five colorectal adenomatous polyps and CRC at 63 and 64 years of age, respectively. A detailed clinical and molecular characterization of this family was previously reported by our group [23].

Among the seven tested index cases found to harbor a PV in the *MUTYH* gene, six were compound heterozygous while one was homozygous for the c.536A>G (p.Tyr179Cys) variant. The identification of biallelic PVs in the *MUTYH* gene molecularly confirmed the autosomal recessive inheritance pattern of MAP in these patients. Segregation analysis of the identified *MUTYH* variants in family members of these seven index cases (families 6–12) revealed that 3 of them carried compound heterozygous PVs, while 30 harbored a monoallelic PV. The c.536A>G (p.Tyr179Cys) variant, identified in a total of five unrelated families, and the c.1187G>A (p.Gly396Asp) variant, detected in two unrelated families, were the most common *MUTYH* PVs occurring in our patient cohort, which is consistent with the observation that they are the most frequent disease-causing *MUTYH* variants [24].

In the whole group of families 6–12, 10 individuals (7 index cases and 3 family members) with biallelic PVs in the *MUTYH* gene developed a variable number of CPs and most often CRC.

**Figure 2:**
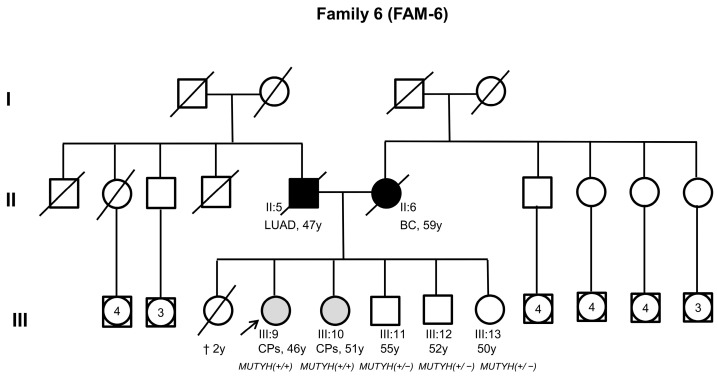
Family pedigree of family 6. Squares indicate men; circles indicate women. Squares and circles with a number inside represent multiple individuals. The arrow indicates the index case. Black-filled symbols denote individuals with cancer, gray-filled symbols indicate individuals with colorectal polyps (CPs), and unfilled symbols correspond to individuals without cancer. Slashed symbols denote dead individuals. The following information is given for specific individuals: clinical manifestations (LUAD = lung adenocarcinoma, BC = breast cancer), age of death (†), age of onset of clinical manifestations, or age at genetic testing (y = years). The zygosity of *MUTYH* pathogenic variants (PVs) is also shown (*MUTHY*+/− = heterozygous PV; *MUTYH* +/+ = compound heterozygous PVs).

In particular, the index case of family 6 (Figure 2, III:9), who was compound heterozygous for the c.325C>T (p.Arg109Trp) and c.1187G>A (p.Gly396Asp) *MUTYH* PVs, developed between 10 and 19 colorectal adenomatous polyps at 46 years of age without a personal or family history of CRC (Figure 2). The same compound heterozygous *MUTYH* PVs were identified in one of the index case’s sisters (Figure 2, III:10), who developed between 10 and 19 colorectal adenomatous polyps at 51 years of age. Instead, the other living siblings of the index case (Figure 2, III:11, III:12, III:13), who only carried a monoallelic *MUTYH* PV (c.325C>T, p.Arg109Trp), did not show clinical manifestations (Table S1).

**Figure 3:**
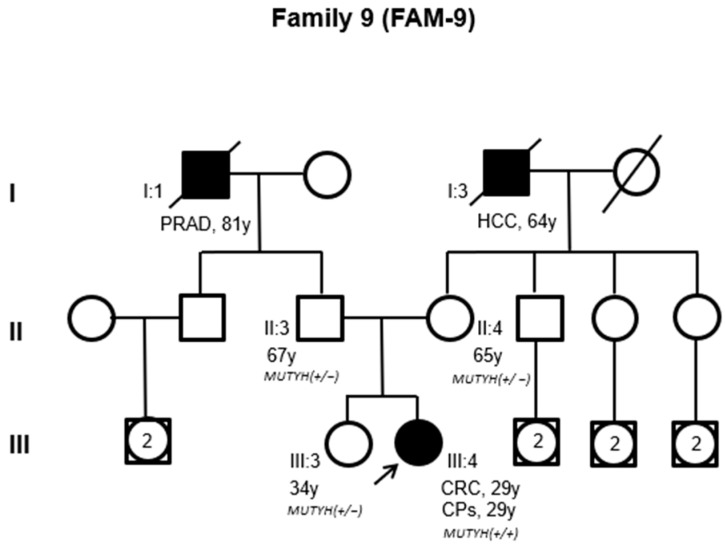
Family pedigree of family 9. Squares indicate men; circles indicate women. Squares and circles with a number inside represent multiple individuals. The arrow indicates the index case. Black-filled symbols denote individuals with cancer, and unfilled symbols correspond to individuals without cancer. Slashed symbols denote dead individuals. The following information is given for specific individuals: clinical manifestations (PRAD: prostate adenocarcinoma; HCC: hepatocellular carcinoma; CRC: colorectal cancer; CPs: colorectal polyps), age of onset of clinical manifestations, or age at genetic testing (y = years). The zygosity of *MUTYH* pathogenic variants (PVs) is also shown (*MUTHY*+/− = heterozygous PV; *MUTYH* +/+ = compound heterozygous PVs).

Similarly, the index case of family 9 (Figure 3, III:4), who harbored the compound heterozygous *MUTYH* PVs c.536A>G (p.Tyr179Cys) and c.884C>T (p.Pro295Leu), developed between 10 and 19 colorectal adenomatous polyps and CRC at 29 years of age. The patient did not have a family history of CPs and CRC. The monoallelic *MUTYH* PV c.884C>T (p.Pro295Leu) was identified in the index case’s unaffected father (68 years of age) (Figure 3, II:3), while the *MUTYH* PV c.536A>G (p.Tyr179Cys) was identified in the index case’s unaffected mother (65 years of age) and sister (34 years of age) (Figure 3, II:4, III:4) (Table S1).

Among the remaining MAP families (families 7, 8, 10–12), seven individuals (five index cases and two family members) with biallelic *MUTYH* PVs developed more than 20 colorectal adenomatous polyps (Table S1).

Based on current NCCN guidelines, the recommended eligibility criterion to genetically test individuals for the presence of PVs in genes (*BMPR1A*, *PTEN*, *SMAD4*, and *STK11*) associated with hamartomatous polyposis syndromes (JPS, PJS, or PHTS) is the occurrence of at least two GI hamartomatous polyps [12].

In family 13, the index case developed one hamartomatous CP at 35 years of age. Genetic testing revealed a *SMAD4* intronic PV (c.425-9A>G, p.Asp142Alafs*7), leading to aberrant *SMAD4* splicing by exonization of intronic nucleotides, which results in a premature stop codon. The same PV was also identified in the index case’s brother, who developed CRC at 45 years of age. The index case and his brother inherited the variant from their mother, who also carried a PV in the *APC* gene (c.3336_3340del, p.Asn1113Serfs*4) and developed CRC and histologically mixed CPs (adenomatous and hamartomatous polyps). This family and the c.425-9A>G (p.Asp142Alafs*7) *SMAD4* variant were characterized clinically and molecularly in a previous study by our group [25].

In family 14, a PV was identified in the *STK11* gene (c.388dupG, p.Glu120Glyfs*33) in a 42-year-old female patient affected by PJS. The index case developed more than two hamartomatous CPs and other CPs histologically classified as sessile tubular adenomas (Table S1). This family was described in detail in a previous report by our group [26].

### 3.3. Clinical and Molecular Findings of Patients with VUSs in Hereditary Polyposis Syndrome-Associated Genes

Among the 75 index cases included in this study, 6 (8%) were identified as harboring 6 different germline VUSs in the *APC*, *MUTYH*, or *BMPR1A* genes (Figure 1a,c and Table S2). In particular, genetic testing revealed that four of these patients (families 15, 16, 19, and 20) who had a personal and family history of CPs and cancer harbored four different germline VUSs involving adenomatous polyposis genes (*APC* and *MUTYH*). Phenotypically, three of these index cases (families 15, 16, and 19) developed less than 10 CPs and CRC, while the index case of family 20 developed between 10 and 19 CPs and extra-colonic cancers (skin cutaneous melanoma, thyroid carcinoma, and prostate adenocarcinoma) (Table S2). Furthermore, the index cases of families 17 and 18 carried two different VUSs in the *BMPR1A* gene. Clinically, these patients had a personal history of CPs and CRC, and a family history of cancer (Table S2). Interestingly, CPs were classified histologically as tubular adenomatous polyps in all the index cases carrying a VUS in the *APC*, *MUTYH*, or *BMPR1A* genes with available histology data (five out of six).

In family 15, a VUS was identified in the *APC* gene (c.5839A>G, p.Thr1947Ala) in a male patient who developed early-onset CRC and CPs (<10) at less than 50 years of age. The index case’s sister carried the same variant and developed an endometrial polyp at 41 years of age (Table S2). Of note, the identified variant was predicted to be disruptive by two in silico tools used to evaluate the effects of missense changes on protein function (Table S3).

Another germinal *APC* VUS (c.6932G>C, p.Arg2311Thr) was identified in the index case of family 16, a 58-year-old male patient who developed two CPs (the first at 53 years of age and the second at 54 years of age) and CRC at 53 years of age (Table S2). Despite the personal and family history of GI cancer of this patient, none of his family members agreed to undergo genetic testing. Interestingly, a probably damaging effect and an alteration in protein function were predicted for the identified VUS by the tools used in our in silico analysis (Table S3).

In family 17, a VUS was found in the *BMPR1A* gene (c.440T>C, p.Phe147Ser) in a 68-year-old male patient with a personal and family history of cancer. However, since his family members declined to undergo genetic testing, we were unable to ascertain if they inherited the variant. The index case developed hyperplastic CPs (<10) and CRC at 68 years of age (Table S2). The identified *BMPR1A* VUS was predicted to be benign by all software used for the in silico prediction analysis (Table S3).

In family 18, another *BMPR1A* VUS (c.485T>G, p.Val162Gly) was detected in a 59-year-old female patient who developed a few (<10) CPs and CRC. The patient had a family history of cancer, and all affected living relatives were screened for the *BMPR1A* VUS. The variant was also identified in the index case’s brother, who developed lung cancer at 20 years of age and a non-Hodgkin lymphoma at 43 years of age, and in one of the index case’s sister, who developed an endometrial polyp at 46 years of age. Another sister developed an esophageal polyp at 55 years of age but did not carry the VUS. The index case’s father had died of CRC at 79 years of age and could not be screened for the variant (Table S2). Based on our in silico analysis, the *BMPR1A* VUS identified in this family was predicted to be probably benign (Table S3).

In family 19, a VUS was detected in the *MUTYH* gene (c.1306C>G, p.Leu436Val) in a female patient who was diagnosed with CRC at 34 years of age. The patient also developed a gastric polyp at 40 years of age and breast cancer 3 years later. Her brother and one of her sisters developed colorectal adenomatous polyps and were found to carry the identified variant. Her father could not be tested for the variant as he had already died of liver cancer (Table S2). In silico prediction analysis indicated that the c.1306C>G (p.Leu436Val) *MUTYH* variant may impair protein function (Table S3).

Another *MUTYH* VUS (c.1493A>G, p.Gln498Arg) was identified in the index case of family 20, a male patient who developed 10–19 CPs between the ages of 50 and 62 years and had a positive family history of cancer. His family members did not agree to undergo genetic testing (Table S2). The c.1493A>G (p.Gln498Arg) *MUTYH* VUS was predicted to be probably benign by the in silico tools used in our analysis (Table S3).

## 4. Discussion

In the present cohort study, we report an investigation of the clinical spectrum and prevalence of germline variants in the main genes associated with hereditary polyposis syndromes in 75 Italian families with CPs that were evaluated as part of genetic counseling at our Institute.

Patients with CPs and their family members, when consenting, underwent molecular analysis of DNA extracted from peripheral blood to identify germline variants in major hereditary polyposis-associated genes (*APC*, *BMPR1A*, *MUTYH*, *PTEN*, *SMAD4*, and *STK11*). This panel of genes was selected in an attempt to find a compromise between the effort to include in the genetic screening all patients with CPs, even those not fulfilling the recommended NCCN 2024 clinical criteria [12], and the purpose to identify PVs allowing us to achieve a specific molecular diagnosis also in patients with early clinical manifestations (lower number of CPs, CPs not well characterized histologically) in order to ensure adequate clinical surveillance and management, while still maintaining screening costs in a reasonable range. Indeed, the identification of PVs in patients with hereditary polyposis syndromes supports the clinical management of their affected family members and has implications for genetic counseling and clinical surveillance [5].

Notably, there is a clear genotype–phenotype relationship for *APC*-associated hereditary CRC syndromes. Based on where the PVs are located on the *APC* gene, four different clinical forms of FAP are described in the literature: (i) classic FAP characterized by extensive polyposis (>1000 polyps), linked to *APC* mutations between codons 1250 and 1424; (ii) AFAP characterized by less than 100 polyps, associated with mutations at the 5′ and 3′ ends of the *APC* gene (before codon 157 and after codon 1595); (3) classic FAP with intermediate colonic polyposis (100–1000 polyps); and GD-FAP characterized by diffuse gastric polyposis, colonic oligopolyposis, and desmoid tumors, and associated with *APC* mutations between codons 2052 and 2843. To date, no other precise genotype–phenotype correlations based on the localization of the causal variant in the remaining target genes (*MUTYH*, *PTEN*, *SMAD4*, and *STK11*) analyzed in this study are known [7].

In our cohort, germline genetic testing for major genes associated with hereditary polyposis syndrome, i.e., *APC*, *BMPR1A*, *MUTYH*, *PTEN*, *SMAD4*, and *STK11*, identified PVs and VUSs in 14 (18.7%) and 6 (8%) index cases, respectively, while no disease-causing genetic variants in the analyzed genes were detected in the remaining 55 (73.3%) patients. Based on these genetic results, we evaluated in detail the clinical phenotypes of the index cases and their familial members carrying variants in these genes.

According to current NCCN guidelines, genetic testing for high-risk genes (*APC*, *MUTYH*) is recommended for individuals with at least 20 colorectal adenomatous polyps and/or a family history of CPs, while it may be considered for individuals with 10–19 colorectal adenomatous polyps [12]. Based on these criteria, in our cohort, the index cases and family members of families 1, 6, and 9 did not fulfill the recommended requirements for genetic testing. However, the results of our molecular screening allowed us to achieve a molecular diagnosis of MAP or FAP for these individuals. It should also be noted that the NCCN guidelines do not provide clear indications about the family history and age of CP and CRC onset (> or <50 years) criteria as a level of evidence for genetic testing in individuals with 10–19 colorectal adenomatous polyps [12]. In this regard, considering the index cases and family members with FAP or MAP in our cohort, two patients of families 6 and 9 developed between 10 and 19 colorectal adenomatous polyps and CRC before 50 years of age, one patient of family 1 developed between 10 and 19 colorectal adenomatous polyps and CRC after 50 years of age in the absence of a family history of CPs and CRC, one patient of family 5 developed less than 10 colorectal adenomatous polyps and CRC after 50 years of age, and one patient of family 6 developed between 10 and 19 colorectal adenomatous polyps after 50 years of age.

Interestingly, in our cohort, genetic testing of patients with CRC and less than 20 colorectal adenomatous polyps allowed the identification of PVs in the main genes (*APC*, *MUTYH*) associated with hereditary polyposis syndromes. Thus, our data indicate that complying with the most stringent recommendation of current NCCN guidelines, which requires the presence of at least 20 colorectal adenomatous polyps, may lead to missing some patients with hereditary forms of polyposis.

As regards hamartomatous polyposis syndromes (JPS, PJS, and PHTS), current NCCN guidelines recommend genetic testing for disease-causing genes (*BMPR1A*, *PTEN*, *SMAD4*, and *STK11*) in patients who develop clinical manifestations that are specific for each syndrome, with the presence of more than two hamartomatous CPs being a common clinical criterion for all of these syndromes [12].

In our cohort, 1 out of 14 (7.14%) index cases carrying a PV received a molecular diagnosis of JPS, and 1 out of 14 (7.14%) received a molecular diagnosis of PJS. Interestingly, a *SMAD4* PV was identified in the index case of family 13 and his family members, although the index case did not meet the classical clinical eligibility criteria for genetic testing for JPS. Thus, our findings indicate that genetic testing of patients who do not completely fulfill the clinical criteria for JPS may be important for detecting PVs in disease-associated genes.

Overall, our study highlights that strict adherence to the clinical criteria guiding patient selection in genetic testing for the molecular diagnosis of hereditary colorectal polyposis may lead to missing some of the patients affected by these syndromes.

Based on current NCCN guidelines, the identification of a VUS cannot be used as a genetic marker to determine cancer risk, and surveillance should be based on family history [12]. In our cohort, 6 out of 75 (8%) tested index cases harbored a VUS. All of these patients had less than 20 colorectal adenomatous polyps. Four index cases had a single VUS in one of the hereditary adenomatous polyposis genes (*APC* and *MUTYH*), and two had a single VUS in the *BMPR1A* gene. The effect of VUSs on gene function and thus on patients’ phenotype and cancer risk is not established [27]. Therefore, further experimental investigations are needed to assess the clinical impact of the VUSs identified in these patients, especially those predicted to be damaging by in silico analysis.

The rate of VUS detection in our cohort is lower than that reported in other cancer patient cohorts screened with larger multigene NGS panels comprising more moderate- and low-penetrance CRC-predisposing genes [28]. Instead, the rate of PVs identified in our patient cohort is consistent with those reported in the scientific literature [28]. As theoretically expected, some clinical features (number of CPs, age of onset CPs or CRC, and the heterogeneity of cancer types) become more attenuated when moving from patients carrying the pathogenic variant to those carrying a VUS. In particular, in 9 out of 14 families (64%), the number of CPs was more than 20 in carrying pathogenic variants (Table S1), unlike families carrying VUS, in which 5 out of 6 families (83%) had less than 10 CPs (Table S2). The age of onset of CPs or CRC is earlier in families carrying PVs than in those carrying VUSs. In particular, in 50% of families with PVs, the age of onset of CPs or CRC was less than 40 years, while only one patient (proband of FAM-19) carrying a VUS developed CRC before the age of 40 years. A final clinical feature found among the subgroups of our cohort of patients is the heterogeneity of cancer types. Families with PVs exhibited reduced heterogeneity of cancer types, including gastric cancer, hepatocellular carcinoma, lung adenocarcinoma, ovarian cancer, and prostate adenocarcinoma, in contrast to patients in the second group with VUSs, who presented a broader spectrum of cancers, such as breast cancer, gastric cancer, hepatocellular carcinoma, non-Hodgkin lymphoma, lung adenocarcinoma, pancreatic adenocarcinoma, prostate adenocarcinoma, skin cutaneous melanoma, and thyroid carcinoma. Moreover, in our study, a large proportion of patients with a personal and/or familial history of CRC and/or CPs remained genetically unexplained. These findings confirmed that highly penetrant genetic causative variants in known predisposing genes (*APC*, *BMPR1A*, *MUTYH*, *PTEN*, *SMAD4*, and *STK11*) are rare and explain only a small fraction of the relative risk in mendelian hereditary polyposis syndromes [29]. In the last decade, different new genes have been identified to be associated with colon polyposis predisposition (*GREM1*, *MBD4*, *MLH3*, *MSH3*, *NTHL1*, *POLD1*, *POLE*, and *RNF43*), each of which explains only a very small proportion of patients with a personal and/or familial history of CRC and/or CPs [29]. Although some of the index cases with negative genetic testing results might be due to lifestyle and environmental factors, it can be hypothesized that other genetic variants in new and different causal genes account for the personal and family history of CRC/CPs in these patients. Additionally, much of the missing heritability observed in the patients and families included in our study may be in part explained by polygenic inheritance, with co-inherited low- or moderate-risk genetic variants being responsible for their family history of CRC [29]. Currently, with the advent of NGS, multiple samples and genes can be sequenced simultaneously more efficiently and cost-effectively than with traditional Sanger sequencing methods. This approach has revolutionized molecular testing in cancer settings, allowing the simultaneous screening of multiple genes associated with genetically heterogeneous disorders. As a result, several susceptibility genes associated with hereditary polyposis syndromes can be tested in a single multigene NGS panel to detect affected patients more quickly and accurately. NGS multigene panels can vary and may include high- or moderate-penetrance CRC susceptibility genes or even genes with a low level of evidence regarding hereditary CRC risk [30].

On one side, the use of NGS multigene panels in clinical practice leads to clinical benefits, such as a higher likelihood of detecting patients with hereditary polyposis syndromes, the possibility to identify co-occurring PVs in different genes related to hereditary CRC syndromes with overlapping clinical presentations, and a better characterization of the clinical manifestations associated with CRC susceptibility genes [23,31]. On the other side, NGS panels encompassing many genes may increase the chance of detecting VUSs or other variants with poor clinical significance in tested patients [27,32]. Therefore, the inclusion of genes in NGS multigene panels designed to aid in the diagnosis of hereditary polyposis syndromes should take into account published evidence supporting gene-disease associations in order to increase the identification of disease-related variants and reduce the detection of genetic alterations that are not characterized molecularly and clinically.

## 5. Conclusions

The present study details our seven-year experience evaluating families with CPs and a history of cancer, expanding our knowledge about the prevalence of PVs in families with polyposis phenotypes. Moreover, it provides insights into the effectiveness of genetic testing of the main genes associated with hereditary polyposis for the screening of patients with one or more relevant phenotypic manifestations.

Based on our experience, an exact correspondence does not always exist between the clinical eligibility criteria for genetic testing recommended in NCCN guidelines and the clinical phenotypes of patients with a suspected hereditary polyposis syndrome (FAP, MAP, PJS, and JPS).

For this reason, the role of clinical geneticists remains crucial in the evaluation of clinical manifestations, age of CP onset, and family history of patients referred to genetic counseling for hereditary polyposis syndromes in order to select those that should undergo genetic testing and choose the most appropriate genes to be included in NGS panels. Moreover, our study highlighted that the identification of a well-known hereditary colon polyposis syndrome can be rarely detected by using multigene panel testing. This could indicate that genetically unsolved patients with a personal and family history of CRC and/or CPs need to be further analyzed using high-throughput DNA sequencing technologies (comprehensive multigene hereditary cancer panels, whole-exome, whole-genome) to potentially identify high-penetrant and low- or moderate-penetrant familial germline variants in other cancer-associated genes to offer tailored clinical surveillance.

## Data Availability

The data presented in this study are available in this article.

## Supplementary Materials

The following supporting information can be downloaded at: https://www.mdpi.com/article/10.3390/cancers16213617/s1, Table S1: Clinical characteristics and family history of index cases with an inherited CRC syndrome carrying a pathogenic variant (PV); Table S2: Clinical characteristics and family history of index cases carrying a Variants of Uncertain Significance (VUS); Table S3: Pathogenicity prediction analysis of the Variants of Uncertain Significance (VUSs) identified in hereditary polyposis-associated genes in our cohort. Figure S1: Protein paint of Pathogenic Variants (PVs) and Variants of Uncertain Significance (VUSs) were identified in our cohort of index cases with colon polyps and a personal/family history of cancer. (a) Distribution of the APC variants (frameshift in red, nonsense in orange, missense in light blue, and splice variants in violet) leading to amino acid changes are marked onto protein domains. Conserved regions and domains of the APC protein are shown. PVs are marked with a red rectangle, and VUSs are marked with a yellow rectangle. (b) Distribution of the MUTYH variants (frameshift in red, nonsense in orange, missense in light blue) leading to amino acid changes are marked onto protein domains. Conserved regions and domains of the MUTYH protein are shown. PVs are marked with a red rectangle, and VUSs are marked with a yellow rectangle. Figure S2: Protein paint of Pathogenic Variants (PVs) and Variants of Uncertain Significance (VUSs) were identified in our cohort of index cases with colon polyps and a personal/family history of cancer. (a) Distribution of the SMAD4 variant (splice variant in violet) leading to amino acid change is marked onto protein domains. Conserved regions and domains of the SMAD4 protein are shown. The identified PV is marked with a red rectangle. (c) Distribution of the STK11 variant (frameshift in red) leading to amino acid changes is marked onto protein domains. Conserved regions and domains of the STK11 protein are shown. The identified PV is marked with a red rectangle. (c) Distribution of the BMPR1A variants (missense in light blue) leading to amino acid changes are marked onto protein domains. Conserved regions and domains of the BMPR1A protein are shown. The identified VUS/s are marked with a yellow rectangle.
