# Supplementary material for: Clinical Assessment and Genetic Testing for Hereditary Polyposis Syndromes in an Italian Cohort of Patients with Colorectal Polyps
Source: Cancers (Basel). 2024 Oct 26;16(21):3617. doi: 10.3390/cancers16213617 (PMC11544946; doi:10.3390/cancers16213617)
Supplement: Supplementary file 1 [file cancers-16-03617-s001.zip › Figure_S1.pptx]

## Slide 1
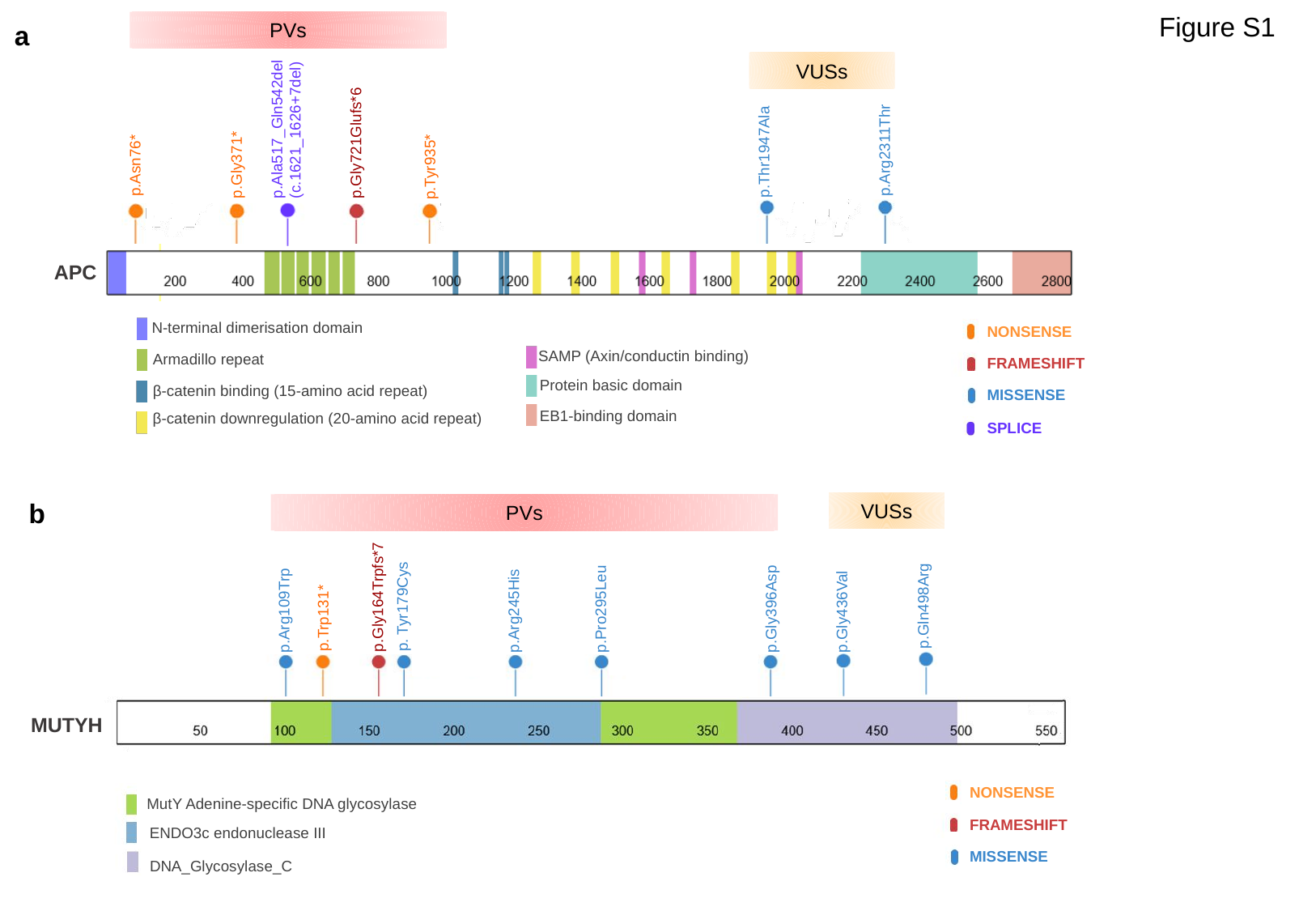

Figure S1
PVs
a
VUSs
p.Ala517_Gln542del
(c.1621_1626+7del)
p.Arg2311Thr
p.Thr1947Ala
p.Gly721Glufs*6
p.Asn76*
p.Gly371*
p.Tyr935*
APC
N-terminal dimerisation domain
NONSENSE
SAMP (Axin/conductin binding)
Armadillo repeat
FRAMESHIFT
Protein basic domain
β-catenin binding (15-amino acid repeat)
MISSENSE
EB1-binding domain
β-catenin downregulation (20-amino acid repeat)
SPLICE
b
VUSs
PVs
p.Gln498Arg
p. Tyr179Cys
p.Gly164Trpfs*7
p.Gly436Val
p.Pro295Leu
p.Gly396Asp
p.Arg245His
p.Arg109Trp
p.Trp131*
MUTYH
NONSENSE
MutY Adenine-specific DNA glycosylase
FRAMESHIFT
ENDO3c endonuclease III
MISSENSE
DNA_Glycosylase_C
