# Supplementary material for: Clinical Assessment and Genetic Testing for Hereditary Polyposis Syndromes in an Italian Cohort of Patients with Colorectal Polyps
Source: Cancers (Basel). 2024 Oct 26;16(21):3617. doi: 10.3390/cancers16213617 (PMC11544946; doi:10.3390/cancers16213617)
Supplement: Supplementary file 1 [file cancers-16-03617-s001.zip › Figure_S2.pptx]

## Slide 1
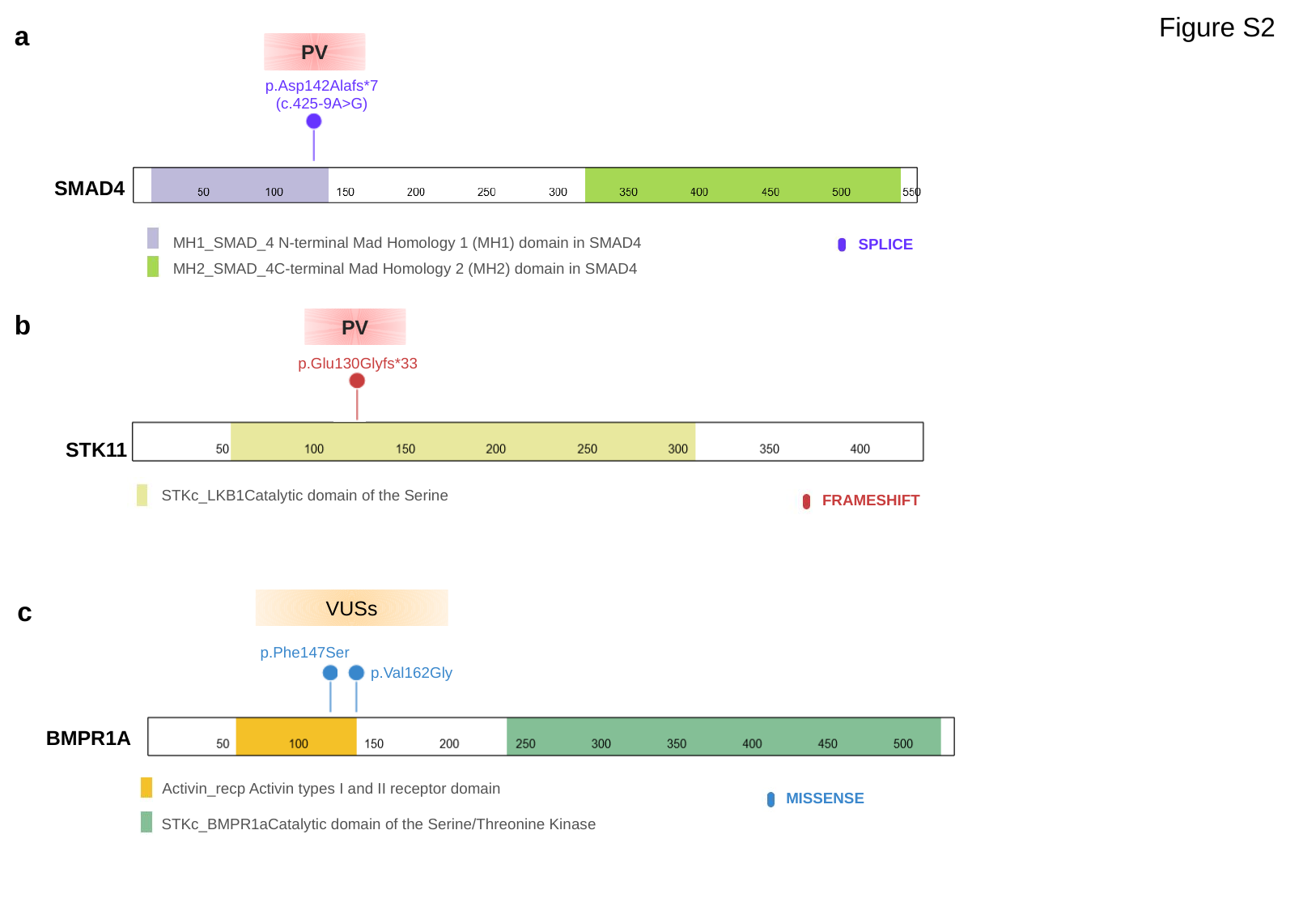

Figure S2
a
PV
p.Asp142Alafs*7
(c.425-9A>G)
SMAD4
MH1_SMAD_4 N-terminal Mad Homology 1 (MH1) domain in SMAD4
SPLICE
MH2_SMAD_4C-terminal Mad Homology 2 (MH2) domain in SMAD4
b
PV
p.Glu130Glyfs*33
STK11
STKc_LKB1Catalytic domain of the Serine
FRAMESHIFT
c
VUSs
p.Phe147Ser
p.Val162Gly
BMPR1A
Activin_recp Activin types I and II receptor domain
MISSENSE
STKc_BMPR1aCatalytic domain of the Serine/Threonine Kinase
