# Supplementary material for: Clinical Assessment and Genetic Testing for Hereditary Polyposis Syndromes in an Italian Cohort of Patients with Colorectal Polyps
Source: Cancers (Basel). 2024 Oct 26;16(21):3617. doi: 10.3390/cancers16213617 (PMC11544946; doi:10.3390/cancers16213617)
Supplement: Supplementary file 1 [file cancers-16-03617-s001.zip › Table_S3.pdf]

Table S3. Pathogenicity prediction analysis of the Variants of Uncertain Significance (VUSs) identified in hereditary polyposis-associated genes in our cohort

Family ID	Gene	Protein change	UniProt ID	Panther Db prediction	Polyphen-2 prediction	SIFT prediction
FAM-15	*APC*	p.Thr1947Ala	P25054	Possibly damaging (Pdel = 0.5)	Benign (score = 0.0)	Affect protein function (score = 0.00)
FAM-16	*APC*	p.Arg2311Thr	P25054	Possibly damaging (Pdel = 0.57)	Probably damaging (score = 0.997)	Affect protein function (score = 0.00)
FAM-17	*BMPR1A*	p.Phe147Ser	P36894	Probably benign (Pdel = 0.13)	Benign (score = 0.002)	Tolerated (score = 0.41)
FAM-18	*BMPR1A*	p.Val162Gly	P36894	Probably benign (Pdel = 0.13)	Benign (score = 0.384)	Affect protein function (score = 0.00)
FAM-19	*MUTYH*	p.Leu436Val	Q9UIF7	Possibly damaging (Pdel = 0.5)	Benign (score = 0.057)	Affect protein function (score = 0.05)
FAM-20	*MUTYH*	p.Gln498Arg	Q9UIF7	Probably benign (Pdel = 0.19)	Benign (score = 0.001)	Tolerated (score = 1.0)
